# Construction of stable packaging cell lines for large-scale industrial BaEV-enveloped retroviral vector production

**DOI:** 10.3389/fimmu.2025.1578660

**Published:** 2025-05-26

**Authors:** Lijun Zhao, Chunting Qiu, Hanyi Chen, Zhuoying Yu, Jiaqi Fan, Qihong Ma, Sijian Zhan, Yaru Feng, Xiaorui Li, Ping Ma, Weijia Wang, Yuanyuan Shi, Jin-fu Xu, Jianxun Wang

**Affiliations:** ^1^ Shenzhen Cell Valley Biopharmaceuticals Co., LTD, Shenzhen, China; ^2^ Department of Infectious Diseases Tianjin Second People’s Hospital, Tianjin, China; ^3^ School of Life Sciences, Beijing University of Chinese Medicine, Beijing, China; ^4^ Guangdong Junhou Biopharmaceuticals Co., LTD, Zhongshan, China; ^5^ Zhongshan City People’s Hospital, Zhongshan, Guangdong, China; ^6^ Department of Respiratory and Critical Care Medicine, Huadong Hospital, Fudan University, Shanghai, China

**Keywords:** BaEV-enveloped retrovirus, stable packaging cell line, industrial-large-scale production, CAR-NK cell therapy, ASCT-1/2 knockout

## Abstract

**Introduction:**

Viral vectors with Baboon endogenous virus (BaEV) envelope proteins have been demonstrated to markedly increase gene transfer efficiency to NK cells. Nevertheless, the cytotoxicity of the BaEV envelope protein necessitates the production of this type of viral vector by transient transfection, which significantly constrains its potential for large-scale industrial application.

**Methods:**

In this study, we constructed a stably packed BaEV-PackRV cell line for BaEVenveloped retroviral vectors. This packaging cell line was constructed to stably express gag, pol, and BaEV envelope proteins, which are essential for retroviral packaging. To this end, we avoided the occurrence of syncytia during virus preparation by knocking out the ASCT-1/2 receptor in the packaging cell line.

**Results and Discussion:**

Compared with the existing methods, the transduction efficiency of the retroviral vector produced by BaEV-PackRV was significantly greater in primary immune cells at a lower multiplicity of infection (MOI), and the transduced CAR-T or CAR-NK cells maintained good expansion capacity and enhanced cytotoxicity. On this basis, our system enables large-scale industrial production of BaEV-coated retroviral vectors while significantly reducing costs.This will greatly improve the efficacy of NK cell gene transfer and the effectiveness of related treatments.

## 1 Introduction

Natural killer (NK) cells were first discovered in the 1970s. As part of the innate immune system, they are distinguished by their capacity to eliminate virus-infected and tumor cells without prior stimulation (1, 2). In recent years, immunotherapy has attracted increasing attention in the field of immunotherapy due to its distinctive immunological characteristics. In combination with genetic engineering, NK cell immune function may be augmented through a range of strategies, including the promotion of cell proliferation (3, 4), the suppression of tumor microenvironment (TME) inhibitory signals, and the enhancement of tumor-specific cytotoxicity (5–7). Although transgenic NK cells have demonstrated certain advantages, there are still some obstacles to their clinical application. Currently, one of the major challenges in NK cell engineering is the efficiency of gene transfer. Despite the greater efficiency of viral vector-based approaches compared to nonviral approaches for transduction, viral vectors based on envelope proteins, such as gibbon ape leukemia virus (GALV) and vesicular stomatitis virus G glycoprotein (VSV-G), remain less efficient for gene transfer in natural killer (NK) cells than in other types of hematopoietic system cells (8–12). The resistance of NK cells to viral transduction may be related to their own intrinsic antiviral mechanisms (13). The use of transduction enhancers, including polybrene and RetroNectin, has been shown to improve the transduction efficiency of viral particles on T and NK cells; however, their cost and potential cytotoxicity have limited their large-scale application (14, 15).

In the context of viral vector production, lentivirus-based vectors are predominantly generated through transient transfection. This method presents several challenges, including low production efficiency, a cumbersome purification process due to the presence of DNA residues in the transfection process, and inconsistencies in viral vector titer and quality across different batches. These shortcomings have resulted in the inability of this method to produce large quantities of viral vectors in a stable manner, thereby limiting its potential for large-scale industrial application. In contrast, retroviral vectors can be produced via the PG13 packaging cell line. This cell line is capable of stably expressing Moloney murine leukemia virus gag-pol proteins and the GALV envelope (16, 17). Following the two-step stable transfection of this cell line with the γ-retroviral backbone encoding the target gene, the corresponding retroviral vectors can be continuously produced in cell culture. This method allows for the stable preparation of large quantities of viral vectors while significantly reducing production costs, and this approach has been widely employed for the industrial production of viral vectors in recent years. The considerable potential of BaEV envelope proteins for gene transfer in NK cells suggests that the production of viral vectors through a combination of BaEV envelope proteins and packaging cell lines will significantly contribute to the advancement of NK cell-based immunotherapy.

**Figure 1:**
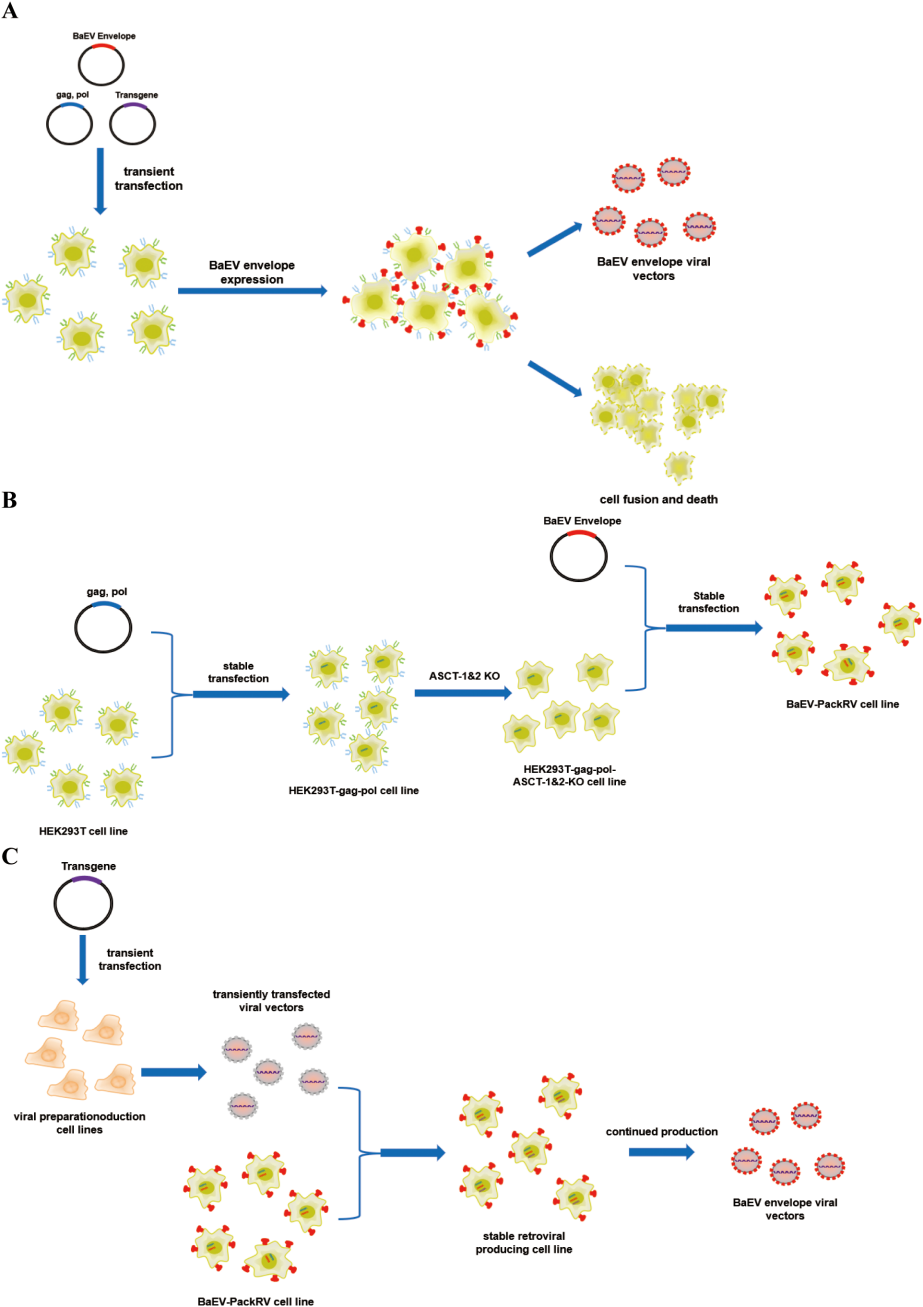
Schematic illustration of the production system for BaEV-enveloped retroviral vectors. **(A)** The production process of BaEV enveloped viral vectors based on transient transfection. **(B)** The construction process of the BaEV-PackRV cell line. **(C)** Two-steps stable-retroviral producing based on BaEV-PackRV cell line.

Baboon endogenous virus (BaEV) is a type of recombinant endogenous gammaretrovirus that is an intermediate between Papio cynocephalus endogenous retrovirus and simian betaretrovirus. BaEV was first isolated in the 1970s and cultured with a human rhabdomyosarcoma cell line (18). Notably, the entry receptor of BaEV is the sodium-dependent neutral amino acid transporter receptor (ASCT-1 and ASCT-2), which is typically highly expressed in the hematopoietic lineage. These findings indicate that BaEV-enriched viral vectors may have broader applications in these cells. Several studies have shown that BaEV-enveloped viral vectors significantly improve the efficiency of gene transfer to CD34+, T, and NK cells in both primary cells and cell lines (19–23). In the case of NK cells, BaEV-enveloped viral vectors provide more than a 20-fold increase in transduction efficiency compared with that of viral vectors generated via the use of VSVG envelope proteins (24, 25). Nevertheless, endogenously produced BaEV envelope proteins can be targeted to cell membranes via ASCT-1/2 receptors, thereby inducing membrane fusion and cell-to-cell fusion (**Figure 1A**). This process is inhibited by the R peptide, which is located at the C-terminus of BaEV envelope proteins. Consequently, envelope proteins lacking the R peptide (BaEV-Rless) have been shown to enhance this process relative to wild-type BaEV (19, 26). This ultimately results in the formation of syncytia and a notable reduction in cell viability (27–29), with ramifications for subsequent viral vector production. The construction of stably packed cell lines based on the BaEV envelope is more challenging due to the presence of this property.

Here, we describe a stably packed cell line (BaEV-PackRV) for BaEV-enveloped retroviral vector production. First, this packaging cell line stably expressed gag, pol, and BaEV envelope proteins. Additionally, to circumvent the influence of BaEV envelope proteins on cellular activity, the gene encoding the ASCT-1/2 receptor was knocked out in this cell line (**Figure 1B**). Furthermore, the retroviral vectors developed based on BaEV-PackRV cell line effectively transduced primary immune cells, especially NK cells. For transient production, only one transfer plasmid needs to be transfected into the BaEV-PackRV cell line. To obtain stable large-scale clinical retroviruses, through the two-step packaging method, the virus encoding the target gene was used to infect the BaEV-PackRV cell line to obtain a stable retrovirus production cell line, after which the retrovirus can be received continuously by expanding the cell population and collecting the cell culture supernatants (**Figure 1C**).

## 2 Materials and methods

### 2.1 Cells and culture conditions

After being resuscitated, the HEK293T cells were cultured in DMEM containing 10% FBS. The cells were incubated at 37°C in a 5% CO_2_ incubator for 48 hours. When the percentage of confluent cells reached approximately 80%, the cells were digested. Thereafter, 8 × 10^5^ cells from each well were inoculated into 6-well plates and subsequently incubated at 37°C in a 5% CO_2_ incubator.

### 2.2 Plasmid transfection

Transfection was subsequently conducted until 70–80% confluence was reached in the HEK293T cells. The transfection system was prepared with 3:1 Fugene HD: DNA and supplemented with 200 μL of DMEM. Following the mixing process, the mixture was allowed to stand at room temperature for 15 minutes, after which it was added dropwise to six-well plates at a ratio of 200 μL per well. The plates were then incubated at 37°C in a 5% CO_2_ incubator.

### 2.3 Drug screening

After 48 h of transduction, the medium was replaced with complete DMEM containing a drug concentration of 3 μg/mL. The cells were treated every two days, and the screening concentration of 3 μg/mL was maintained. After one week of drug screening, the cells were treated every four days, and the drug concentration was increased to 8 μg/mL.

### 2.4 Monoclonal selection

Following the digestion of drug-screened cells, the cell density was adjusted to 100 cells/mL, and the cells were inoculated into 96-well plates. A volume of 100 µL of cell suspension was added to each well, and the plates were incubated at 37°C in a 5% CO_2_ incubator. The growth status of the cells was monitored, and the monoclonal cells were gradually expanded and frozen.

### 2.5 Real-time PCR assay

The primers used for gag-pol gene detection were gag-pol-qPCR-F:GACGGAAATGGTGGAGA; gag-pol-qPCR-R:ATGCCTGCGAGGTAGTG. The primers for BaEV envelope detection were BaEV-qPCR-F:GGCATACACTTATCTACCCACGAAC; BaEV-qPCR-R:TGATCAATAGCAGGGATGGGGAC. RNA was extracted from monoclonal cells and subsequently reverse-transcribed to obtain cDNA. For real-time fluorescent quantitative PCR, 10 μL of 2× SYBR mix, forward primer (0.25 μM), reverse primer (0.25 μM), and 5 μL of cDNA template were added to each PCR tube. DNase/RNase-free water was added to bring the volume to 20 μL. The thermal cycling conditions included 95°C for 5 min, followed by 40 cycles of 95°C for 10 s and 60°C for 30 s.

### 2.6 Viral vector production and titer assay

The virus-producing cells were incubated at 32°C in a 5% CO_2_ incubator, and the supernatant was collected after 48 hours. The supernatant was filtered through a 0.45 μm filter membrane and subsequently subjected to a titer assay.

For the titer assay, 2×10^5^ HEK293T cells were inoculated in 24-well plates. Following 24 hours of incubation, the medium was removed, and 1 mL of the virus mixture to be tested, along with 1 μg/mL polybrene, was added. The cells were subsequently subjected to centrifugation at 2500 rpm at 32°C for one hour, after which they were incubated at 37°C in a 5% CO_2_ incubator. Following 24 hours of viral transduction, the medium was replaced with fresh medium. After 48 hours, the transduction efficiency was detected via flow cytometry. The viral biological titer was calculated according to the following formula: viral titer (TU/mL) = number of cells at the time of infection * percentage of positive cells/volume of viral stock solution.

### 2.7 Construction of the ASCT-1&2 knockout plasmid

The ASCT-1-gRNA and ASCT-2-gRNA fragments were designed in accordance with the gene sequences (**Supplementary Table S3**). The target fragments were formed by primer self-association. The PX458 plasmid was double digested with BbsI restriction endonuclease, and the product was recovered. The target fragment and linearized vector were subjected to Gibson Assembly. The products were transformed into competent DH5α cells and verified via PCR and sequencing.

### 2.8 Electroshock transformation

The plasmid was added to 150 μL of electrotransformation buffer and prepared as an electrotransformation reaction mixture. A total of 1×10^6^ cells were collected and centrifuged, and the supernatant was discarded. The electrotransformation reaction mixture was then added, and the cells were gently aspirated and transferred to the electrotransformation cup. Electrotransformation was performed via the Q-001 program on a Lonza 2b electrotransferometer. Finally, the cell suspension in the electrotransfer cup was aspirated and added to prewarmed DMEM. Following 24 hours of incubation, the GFP-positive cells were sorted into single-cell clones via flow cytometry.

### 2.9 Knockout efficiency assay

The knockout effect of ASCT-1 and ASCT-2 was determined by comparing the sequencing results from different monoclonal cells with the genome sequences. The knockout effect of ASCT-1 was determined using DECODR (DNA Editing Analysis Software, https://decodr.org), while the sequence comparison of ASCT-2 was performed using SnapGene software.

### 2.10 PCR detection

The genome of monoclonal cells was amplified with three paired primers to verify the knockout of the ASCT-2 gene (1-ASCT-2-F/R: CTTTCGCTCAGTGAGTCCTC/CACAGCAAAGACTAAGGCAG; 2-ASCT-2-F/R: GAGAGCATGAGATTGGCATG/GAATCTCCTGAAGTATGGCC; 3-ASCT-2-F/R: CTCTCTTGGTTCCCACACTC/GGTCATCTGGCTCCAAAGAG). The resulting amplification products were then subjected to agarose gel electrophoresis for detection.

### 2.11 Monoclonal cell growth curves

A total of 4×10^5^ monoclonal cells were harvested and inoculated into six-well plates, and HEK293T cells were used as a control. Cell counts were performed every two days to plot growth curves.

### 2.12 Construction of stable retrovirus-producing cell lines

The virus-producing cell lines were transfected with the pMFG transfer plasmid carrying the target gene, and the cell supernatants were collected to harvest transiently transfected retroviral vectors. The supernatant obtained in the first step was then mixed with the BaEV-PackRV cells, and transduction of the retroviral vectors was facilitated by horizontal centrifugation to obtain a stable retrovirus-producing cell line. If the transduction rate exceeded 50%, a stable retrovirus-producing cell line was obtained. When the degree of cell fusion reached 90%, the virus-producing cells were incubated at 32°C in a 5% CO_2_ incubator, high-titer retrovirus vectors were obtained by harvesting the culture supernatant for 4 continuous days. The production of viral vectors in volumes of 50 mL, 500 mL, and 1 L is conducted in a facility that adheres to good manufacturing practice (GMP) standard with the same parameters.

### 2.13 T-cell and NK-cell transduction

First, RetroNectin (10 μg/mL) was added at 1 mL per well to a 12-well plate (no tissue treatment) and protected from light at 4°C overnight. The next day, the RetroNectin solution was removed, and the well plates were rinsed with PBS. Subsequently, 1 mL of fresh retroviral vector was added to the well plate, which was subsequently centrifuged at 32°C and 2500 rpm for 1 hour. After removal of the supernatant, 4×10^5^ activated T cells or NK cells were resuspended in 1 mL of fresh retroviral vector, and polybrene (final concentration of 6 μg/mL) was added to each well. The 12-well plate was centrifuged at 32°C and 2500 rpm for 1 hour. The cells were subsequently transferred to a 37°C incubator for 2 hours. The above steps were repeated if necessary. After 2 and 8 days of transduction, the transduction efficiency was detected via flow cytometry.

### 2.14 Cytotoxicity assay

For the luciferase assay, Raji-luc cells were cocultured with CAR-NK cells or NK cells at E/T ratios of 8:1, 4:1, 2:1, 1:1, 1:2, 1:4, or 1:8 for 12 hours in a 96-well white assay plate (Corning, NY, USA) (0.1 mL/well). Next, 100 µL of Luciferase Assay Reagent was added, and the cells were incubated at room temperature for 5 minutes. The fluorescence was measured on a SpectraMax Series Multi-Mode Microplate Reader, and the data were analyzed via SoftMax Pro 6.4.2 software (Molecular Devices). Lysis was calculated according to the following formula: % lysis = (experimental lysis − spontaneous lysis) × 100/(maximum lysis − spontaneous lysis).

## 3 Results

### 3.1 Construction of the HEK293T-gag-pol cell line

**Figure 2:**
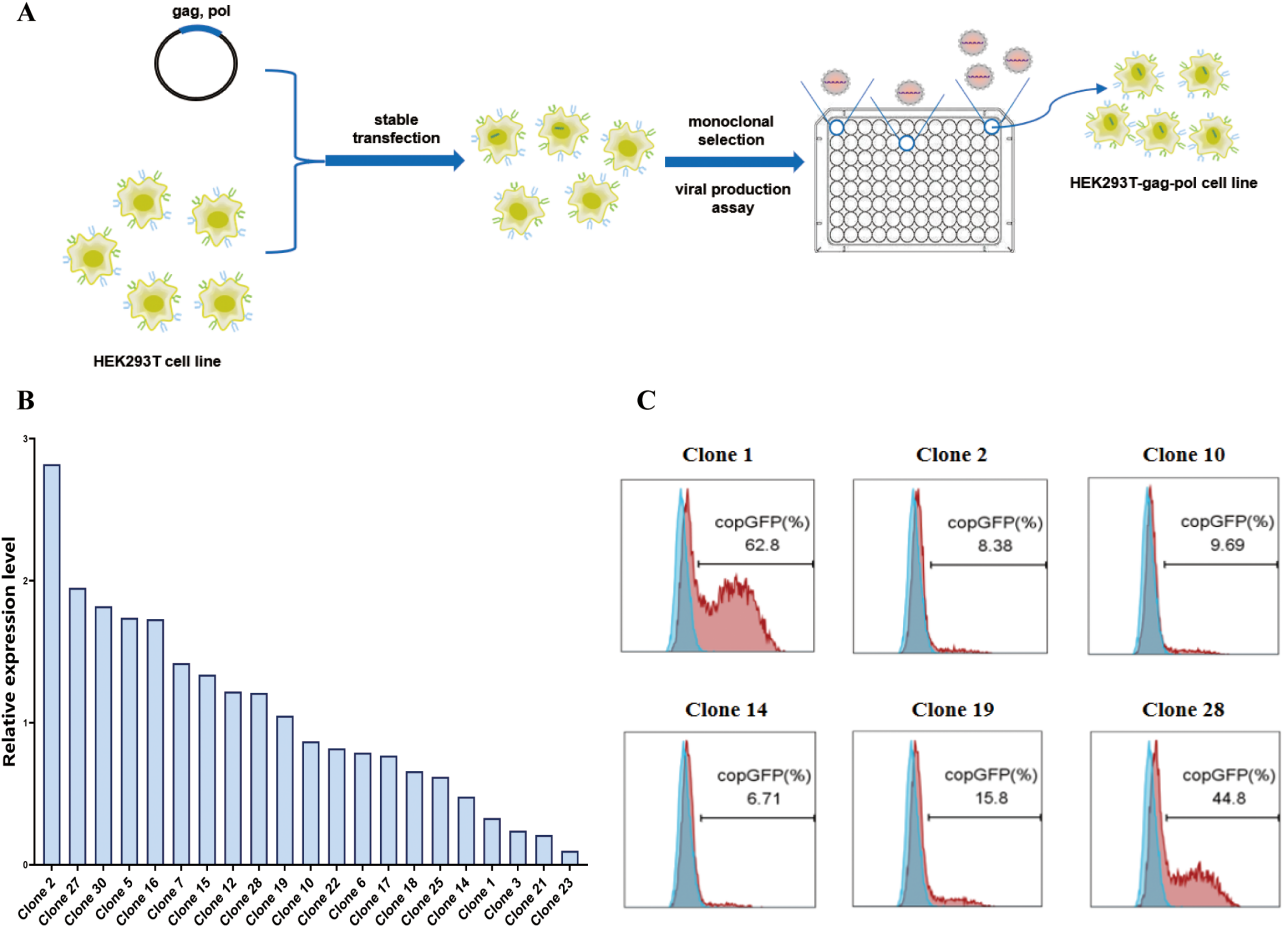
Construction of the HEK293T-gag-pol cell line. **(A)** Schematic drawing of the construction process. **(B)** Relative expression level of the gag-pol gene in HEK293T-gag-pol monoclonal cells. **(C)** Flow cytometry detection of the viral titer produced by each HEK293T-gag-pol monoclonal cell line.

First, we used the pCMV-MMLV-gag-pol-PGK-puro plasmid to transfect HEK293T cells. The transfected cells were subsequently subjected to puromycin screening and monoclonal selection(**Figure 2A**). As indicated by the results of real-time fluorescent quantitative PCR (**Figure 2B**), a total of six monoclonal cells exhibiting high (clone 2), medium (clone 10, clone 19, clone 28), and low (clone 1, clone 14) expression of the gag-pol gene were selected for subsequent transient transfection and retroviral production assays (**Figure 2C**, **Supplementary Table S1**). In order to verify the retroviral production ability of different monoclonal cells, the monoclonal cells were subjected to transfection with the pMFG-copGFP transfer plasmid and the wild-type BaEV envelope plasmid (pCMV-BaEV-WT-PGK-hygr) for retroviral production. This process produces retroviral particles with copGFP as a reporter gene. Subsequent to transduction of HEK293T cells, the retroviral biological titer can be reflected by percentages of GFP+ cells. HEK293T-gag-pol clone 1, which presented the highest viral titer, was selected for subsequent construction. This finding also reflects the fact that the gag-pol gene copy number does not correlate with the eventual ability to produce retroviruses.

### 3.2 Construction of the HEK293T-gag-pol-ASCT-1&2-KO cell line

**Figure 3:**
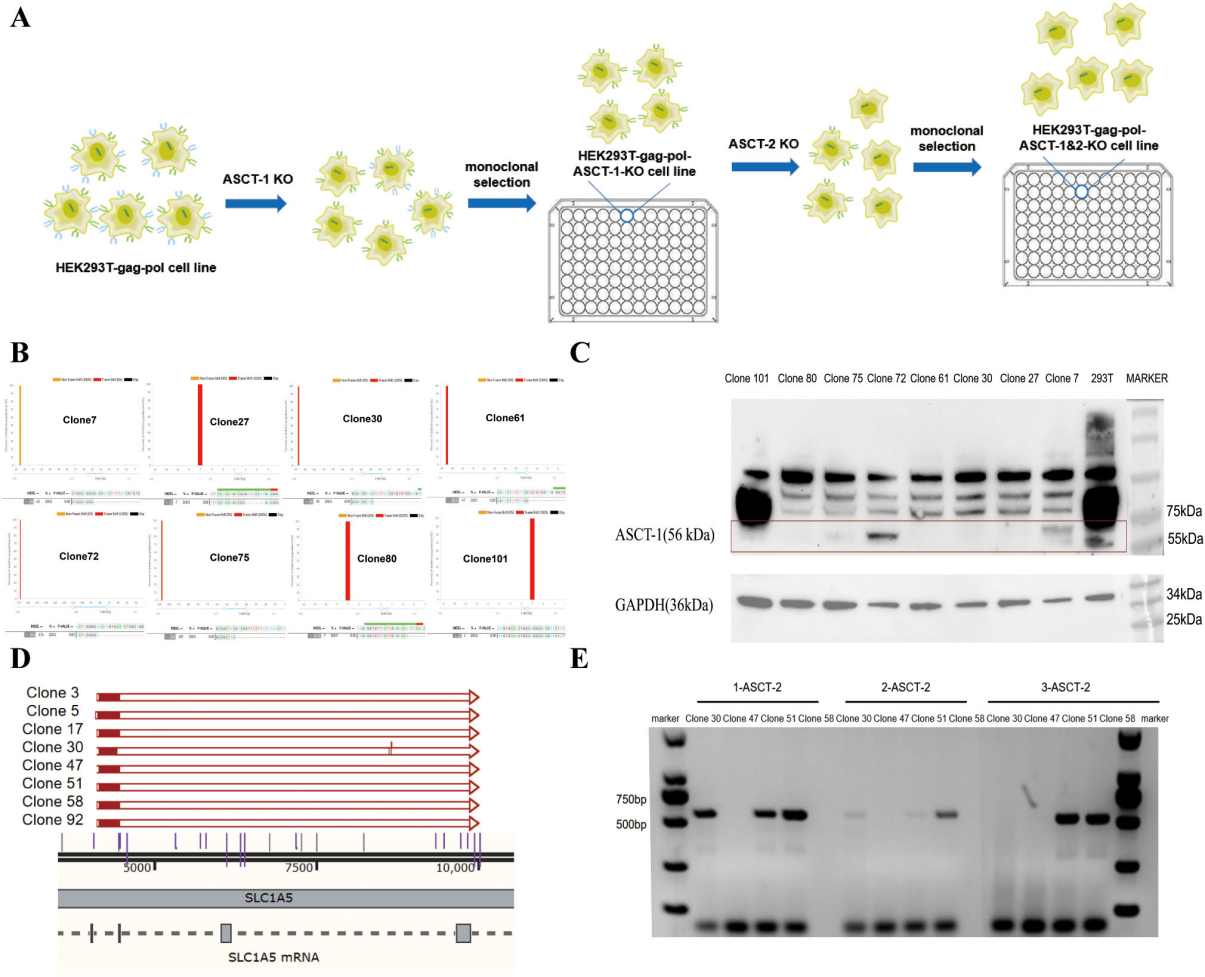
Construction of the HEK293T-gag-pol-ASCT-1&2-KO cell line. **(A)** Schematic drawing of the construction process. **(B)** Sequencing identification of ASCT-1 knockout monoclonal cells. **(C)** Western blot validation of ASCT-1 expression in knockout monoclonal cells. **(D)** Sequencing identification of ASCT-2 knockout monoclonal cells. **(E)** PCR detection of ASCT-2 knockout monoclonal cells.

During virus production, the BaEV-WT envelope protein induced severe syncytium formation, resulting in the rapid death of HEK293T cells unable to produce virus, thus reducing the viral titer. To address this, we used genome editing technology to knock out both the ASCT-1 and ASCT-2 genes in HEK293T cells, thereby eliminating BaEV-induced cell fusion (**Figure 3A**). To construct ASCT-1&2 knockout cell lines, HEK293T-gag-pol clone 1 cells were first electrotransfected with the PX458-ASCT-1-gRNA-GFP plasmid carrying Cas9 protein (SpCas9) and gRNA (**Supplementary Figure S1**), and sorted into clones that did not express ASCT-1. Eight single-cell clones were selected and identified via Western blotting and sequencing to verify the knockout efficiency (**Figure 3B**). (Primer: ASCT-1-F: GGTCTGAGACAAGACACATG; ASCT-1-R: GTCACTCTAAGGGATGGCAG). Western blot analysis demonstrated that the ASCT-1 protein was not expressed in HEK293T-gag-pol-ASCT-1-KO clone 30 (**Figure 3C**). Based on these results, clone 30 was selected for subsequent ASCT-2 gene knockout.

To knock out both exons of the ASCT-2 gene, HEK293T-gag-pol-ASCT-1-KO clone 30 was electrotransformed with the PX458-ASCT-2-gRNA-1 plasmid and the PX458-ASCT-2-gRNA-2 plasmid. After a new round of monoclonal cell screening, eight single-cell clones with deletion fragments exceeding 5000 bp were subjected to sequencing to ascertain the complete destruction of all ASCT-2 protein transcripts (**Figure 3D**). Clones 30, 47, 51, and 58 were subsequently chosen for further PCR validation experiments with three paired primers (**Figure 3E**). The three pairs of primers were designed with the specific intention of amplifying deletion fragments. Theoretically, the absence of amplification products indicates a complete knockout. The findings indicated that only clone 47 exhibited a negative response to all three primer pairs, thus confirming the efficacy of the ASCT-2 gene knockout in the monoclonal cells of this particular clone. Consequently, clone 47 with knockout of both ASCT-1 and ASCT-2, named HEK293T-gag-pol-ASCT-1&2-KO cells, was selected for subsequent construction.

### 3.3 Construction of the HEK293T-gag-pol-ASCT-1&2-KO-BaEV cell line

**Figure 4:**
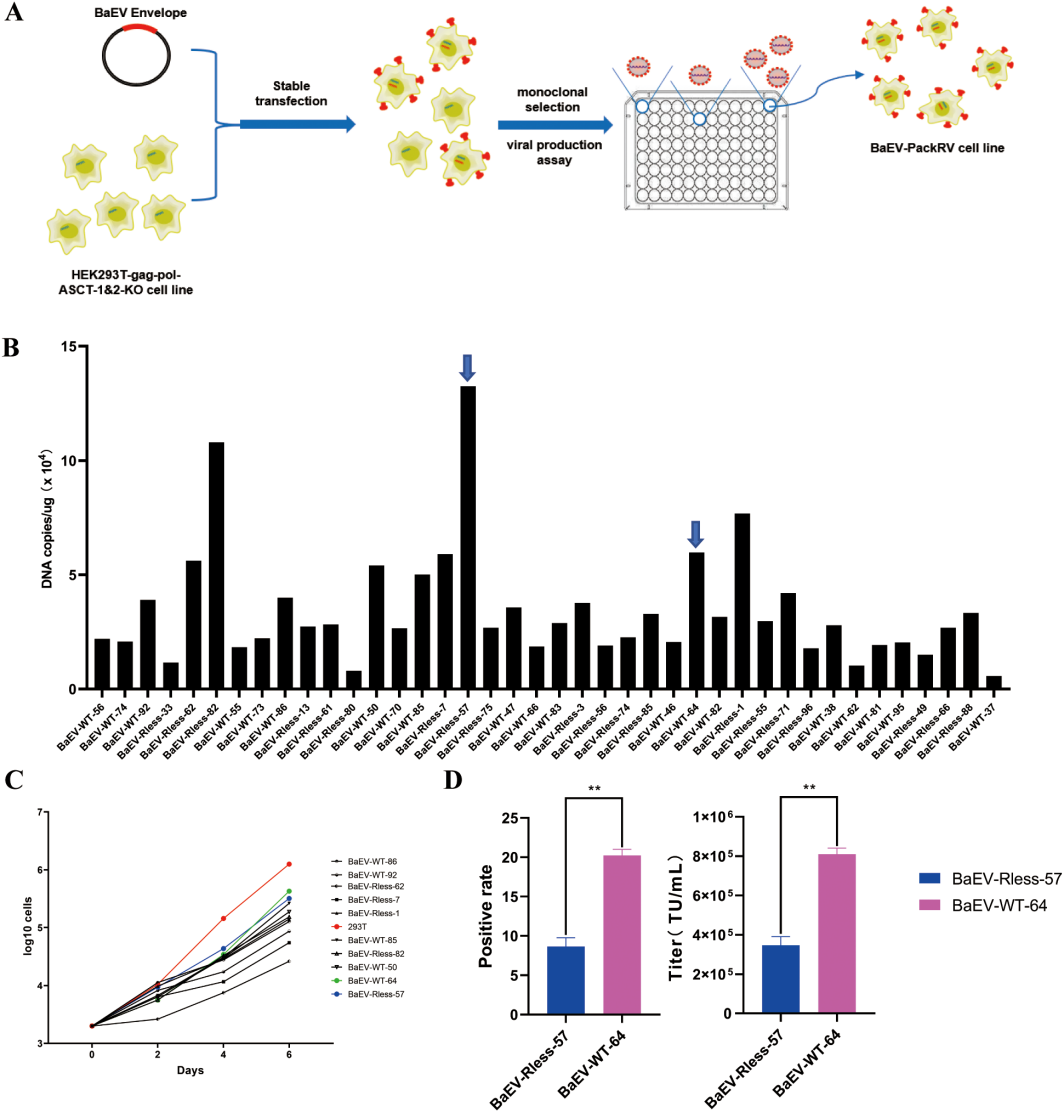
Construction of the HEK293T-gag-pol-ASCT-1&2-KO-BaEV cell line. **(A)** Schematic drawing of the construction process. **(B)** RT–PCR detection of BaEV-WT or BaEV-Rless copy numbers in monoclonal cells. **(C)** Growth curve of monoclonal cells. **(D)** Flow cytometry detection of the viral titer produced by BaEV-Rless-57 or BaEV-WT-64 monoclonal cells, The values shown are the average of three independent experiments and their standard errors (**, p<0.01).

We further constructed the above cell lines to stably express the BaEV envelope (**Figure 4A**). HEK293T-gag-pol-ASCT-1&2-KO cells were transfected with a wild-type BaEV plasmid (pCMV-sBaEV-WT-PGK-hygr) or BaEV-Rless plasmid (pCMV-BaEV-Rless-PGK-hygr). The growth status of both cell lines was recorded by microscopy 24 hours after transfection (**Supplementary Figure S2**). The cellular status of the HEK293T-ASCT-1&2-KO cells was significantly superior to that of the wild-type HEK293T cells after transfection. Transfection with the BaEV-WT or BaEV-Rless plasmid alone also induced severe syncytium formation in HEK293T cells, while BaEV-induced cell fusion was abolished in HEK293T-gag-pol-ASCT-1&2-KO cells. This result suggested that knockout of the ASCT-1 and ASCT-2 genes was necessary and resolved the problem of syncytium formation after BaEV-WT or BaEV-Rless transfection, which enabled normal viral production.

After hygromycin screening and monoclonal selection, the expression level of the BaEV-Rless or BaEV-WT envelope was detected via real-time fluorescent quantitative PCR (**Figure 4B**). Thus, monoclonal cells with different copy numbers of the BaEV-WT or BaEV-Rless envelope were selected and subjected to growth curve detection. The results of the growth curves demonstrated that the proliferation time of all monoclonal cells was prolonged in comparison with that of wild-type HEK293T cells (**Figure 4C**). Based on the growth curves, the BaEV-WT-64 clone and BaEV-Rless-57 clone, which presented slightly faster growth rates, were selected for transient transfection and viral production assays. To assess viral production capacity, BaEV-WT-64 and BaEV-Rless-57 monoclonal cells were transfected with the pMFG-copGFP plasmid for retroviral production. The supernatants containing retrovirus were collected 48 hours after transfection, and the viral titer was determined by flow cytometry (**Figure 4D**). The virus produced by BaEV-WT-64 clone cells presented a higher viral titer than the virus produced by BaEV-Rless-57 clone cells.

At present, the cell line has been completely constructed for BaEV-WT or BaEV-Rless-pseudotyped retrovirus (BaEV-WT-RV or BaEV-Rless-RV) production, and it stably expresses the gag-pol gene and envelope gene; thus, only one transfer plasmid needs to be transfected during retrovirus production, simplifying the transient transfection process. Transient transfection of the pMFG-copGFP transfer plasmid confirmed that the cell line could successfully package retrovirus without the formation of syncytia. A stable transduction-viral production assay was subsequently conducted.

### 3.4 Construction of a BaEV-copGFP-RV stable retrovirus-producing cell line

**Figure 5:**
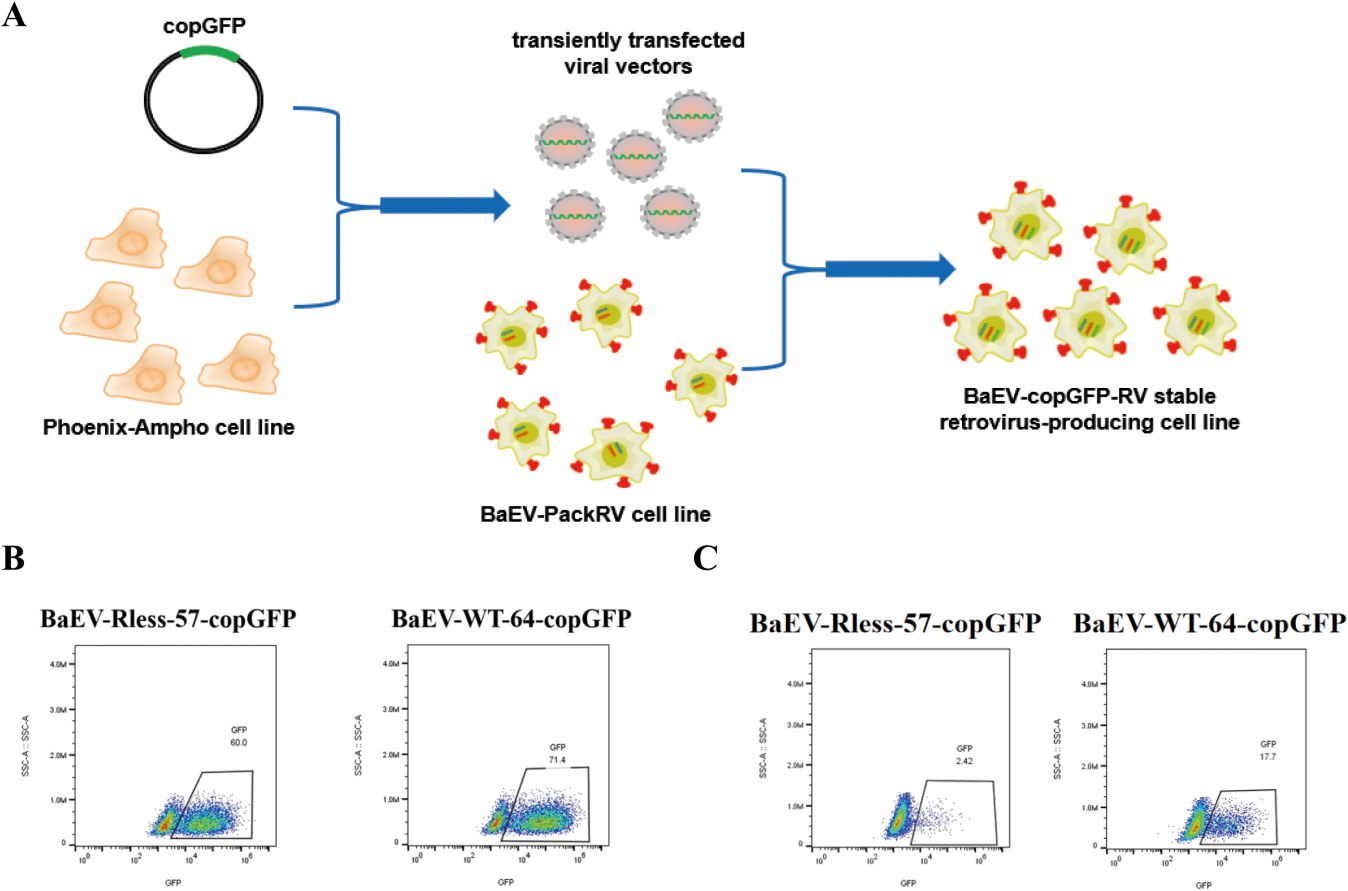
Construction of a BaEV-copGFP-RV stable retrovirus-producing cell line. **(A)** Schematic drawing of the construction process. **(B)** Flow cytometry analysis of the transduction rate of BaEV-Rless-57-copGFP and BaEV-WT-64-copGFP retrovirus-producing cells. **(C)** Flow cytometry detection of the retroviral titer produced by stable BaEV-Rless-57-copGFP- and BaEV-WT-64-copGFP-transduced cells.

The retroviral vectors were generated via a two-step method using Phoenix-Ampho and BaEV-packaging cell lines as described above, similar to the Phoenix-ECO and PG13 cell lines, respectively (**Figure 5A**) (24, 25). In this study, the pMFG-copGFP transfer plasmid was initially transfected into Phoenix-Ampho cell lines or other retrovirus preparation cell lines. The supernatant obtained in the first step was then transduced into BaEV-Rless-57 or BaEV-WT-64 monoclonal cells until the transduction rate exceeded 50%, thereby generating stable BaEV-copGFP-RV-producing cell lines. A flow cytometry assay demonstrated that the positive transduction rates of both BaEV-Rless-57-copGFP cells and BaEV-WT-64-copGFP cells exceeded 60% (**Figure 5B**), thus allowing for the preparation of the retrovirus. We collected the culture supernatant and detected the virus titer. The results of the titer assay demonstrated that the titer of the retroviral vector produced by BaEV-WT-64-copGFP cells was greater than that produced by BaEV-Rless-57-copGFP cells (**Figure 5C**, **Supplementary Table S2**). Consistent with the trend of the retroviral titer in the transient transfection assay (**Figure 4E**), wild-type BaEV was better for retrovirus packaging. Following the deletion of the R peptide sequence inhibiting cell-to-cell fusion from the C-terminus of the wild-type BaEV gp, it was observed that BaEV-Rless exhibited a stronger fusogenic activity. Consequently, this would enhance the invasive capacity of the viral vector to a certain degree. However, it is important to note that the copy number and stability of BaEV gp in the viral envelope also affect the viral titer. In lentiviral vector-based viral production systems, BaEV-Rless has been shown to exhibit higher titer than wild-type BaEV gp (19, 26). However, the results of this study suggest that wild-type BaEV gp may be more suitable for γ-retroviral-based viral production systems.

Based on the outcomes of transient transfection and stable transduction tests, BaEV-WT-64 monoclonal cells were identified as the optimal BaEV-retroviral vector packaging cell line for subsequent use, and the BaEV-PackRV cell line was also named for patent application and cell preservation. This cell line can be used as a transient transfection cell line, or it can also be transformed into a stable retrovirus-producing cell line like PG13 via a two-step method.

### 3.5 BaEV-CD19 CAR-RV showed comparable transduction efficiency in T cells

**Figure 6:**
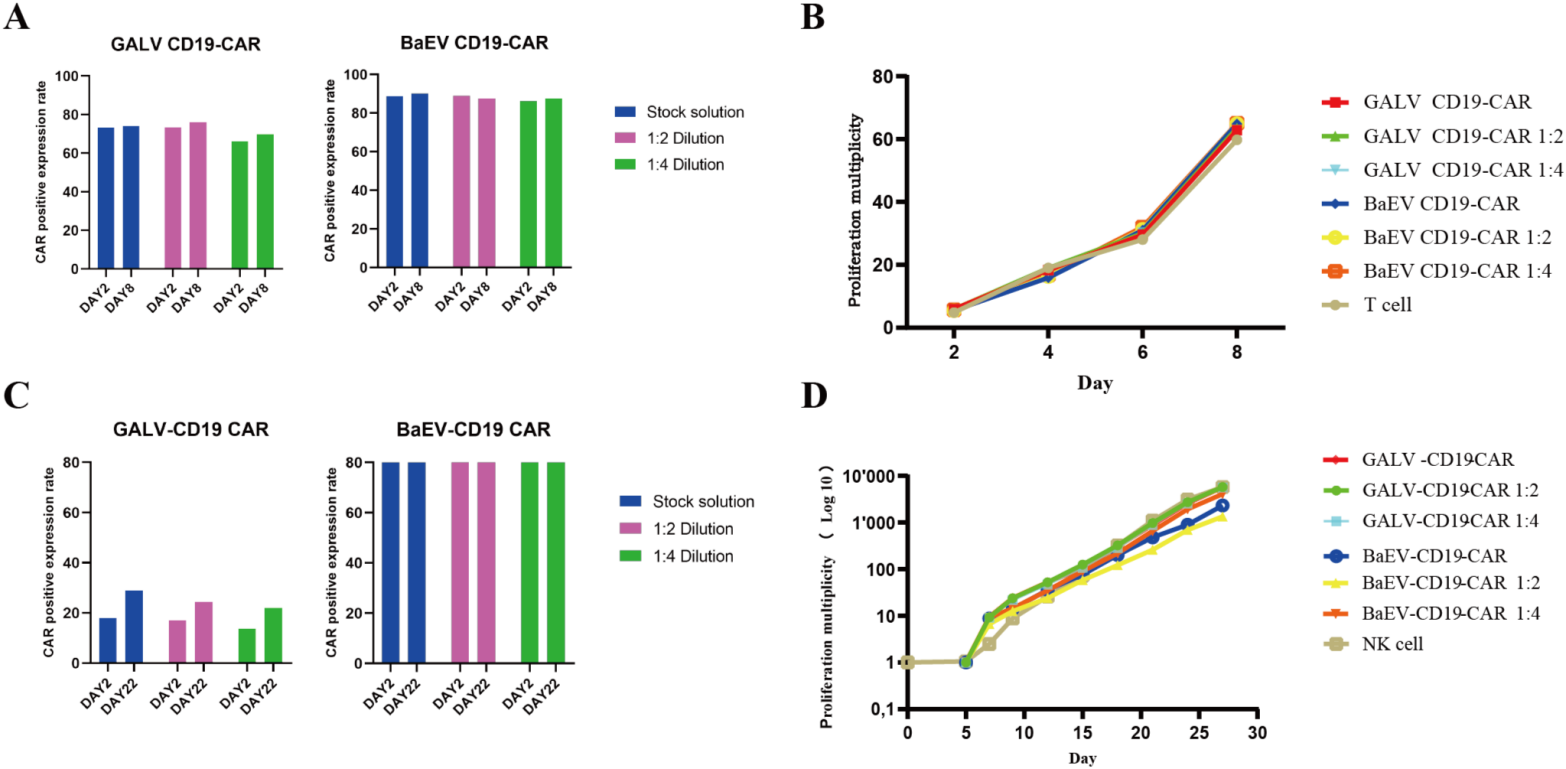
BaEV-CD19 CAR-RV and GALV-CD19 CAR-RV comparisons. CD19 CAR-T-cell **(A)** and CD19 CAR-NK cell **(C)** CAR-expressing levels were detected by flow cytometry after transduction with BaEV-CD19 CAR-RV or GALV-CD19 CAR-RV. CD19 CAR-T-cell **(B)** and CD19 CAR-NK cell **(D)** proliferation rates after transduction.

Next, we directly compared two stable retroviral packaging cell lines: the GALV-pseudotyped MLV-producing cell line PG13 and the BaEV-pseudotyped BaEV-PackRV cell line. Based on the aforementioned two-step stable retroviral production method, we separately prepared BaEV-CD19 CAR-RV and GALV-CD19 CAR-RV. The transduction efficiency of BaEV-CD19 CAR-RV to T cells and NK cells was evaluated, with GALV-CD19 CAR-RV serving as a control. Although the retrovirus titer of BaEV-CD19 CAR-RV was lower than that of GALV-CD19 CAR-RV, the T-cell transduction efficiency was greater than that of GALV-CD19 CAR-RV when either the retrovirus stock solution or the diluted virus was used (**Figure 6A**, **Table 1**). Even if the retrovirus vector was diluted 4 times, the percentage of CD19 CAR-T cells transduced with BaEV-CD19 CAR-RV was maintained at the same level as that of the stock medium, indicating that BaEV-CD19 CAR-RV still had good transduction efficiency for T cells at very low titers and low MOI(multiplicity of infection, the ratio of the virus to the cells at the time of transduction). The transduction efficiency of GALV-CD19 CAR-RV was obviously reduced at 1:4 dilution. Moreover, the positive rate of CD19 CAR-T cells did not change significantly at day 2 or day 8 after transduction, indicating that both GALV- and BAEV-pseudotyped RVs were stably expressed. CD19 CAR-T-cell proliferation analysis indicated that there was no significant difference between BAEV-CD19-CAR-RV transduction and GaLV-CD19-CAR-RV transduction (**Figure 6B**). These findings suggested that BaEV-CD19 CAR-RV significantly enhanced the transduction efficiency of primary T cells without affecting CD19 CAR-T-cell proliferation.

**Table 1:** CD19 CAR-T cell CAR-positive expression rate.

Type of retroviral vector	RV Titer TU/mL	Detection time (after transduction)	Stock solution	MOI	1: 2 Dilution	MOI	1: 4 Dilution	MOI
GALV CD19-CAR	3.9 E+06	DAY2	73.2%	9.75	73.4%	4.88	66.1%	2.44
		DAY8	74.1%		76.1%		69.9%	
BaEV CD19-CAR	1.38 E+06	DAY2	88.7%	3.45	88.9%	1.73	86.2%	0.86
		DAY8	90%		87.6%		87.5%	

### 3.6 Compared with other retroviruses, the BaEV-CD19 CAR-RV showed excellent transduction efficiency in NK cells

Genetic engineering of NK cells remains challenging and requires optimized viruses and protocols. Compared with primary T cells, primary NK cells are more difficult to transduce, and the CAR-positive rate of most CAR-NK studies can reach only 30-40% with either lentivirus or retrovirus (30). In our study, despite a lower titer than that of GALV-CD19 CAR-RV, the CAR-positive transduction rate of BaEV-CD19 CAR-RV on NK cells was markedly higher than that of GALV-CD19 CAR-RV. Across all MOIs, the percentage of BaEV-CD19 CAR-NK cells reached 82.7-90.3% (**Figure 6C**, **Table 2**). Following fourfold dilution, the transduction efficiency of the BaEV-CD19 CAR was 82.7% at day 2 and 84.5% at day 22, whereas the positive transduction rates of the GALV-CD19 CAR were only 13.8% and 22.1%, respectively. These results demonstrated that BaEV-RV and our transduction protocol markedly enhanced the transduction efficiency of primary NK cells and that the CAR expression level was stable. The results of CAR-NK cell proliferation analysis up to day 22 after transduction revealed minimal differences between the BAEV-CD19 CAR-RV and GaLV-CD19 CAR-RV groups (**Figure 6D**). These findings suggested that BaEV-CD19 CAR-RV significantly enhanced the transduction efficiency of primary NK cells without affecting CD19 CAR-NK cell proliferation.

**Table 2:** CD19 CAR-NK cell CAR-positive expression rate.

Type of retroviral vector	RV Titer TU/mL	Detection time (after transduction)	Stock solution	MOI	1: 2 dilution	MOI	1: 4 dilution	MOI
GALV-CD19 CAR	3.9 E+06	DAY2	17.9%	9.75	17%	4.88	13.8%	2.44
		DAY22	29%		24.5%		22.1%	
BaEV-CD19 CAR	1.43 E+06	DAY2	90.3%	3.58	90.9%	1.79	82.7%	0.89
		DAY22	93%		92.2%		84.5%	

**Figure 7:**
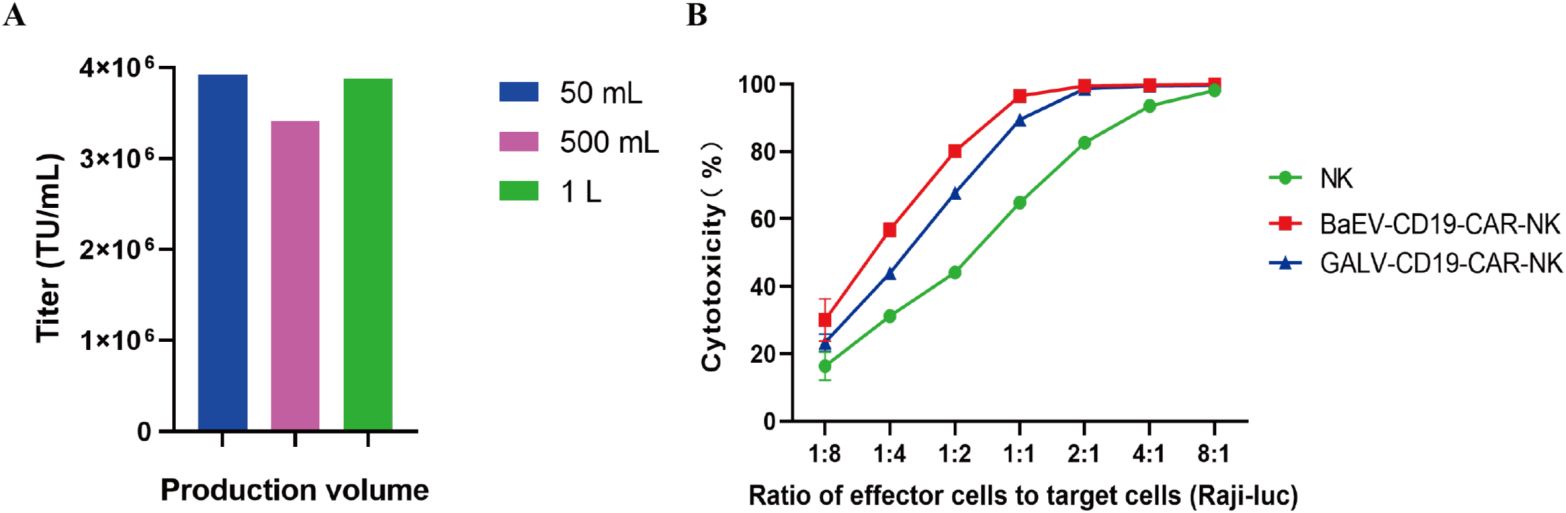
Titer and functional validation of BaEV-CD19 CAR-RV produced in large volumes. **(A)** The titer of BaEV-CD19 CAR-RV produced at the industrial scale. **(B)** Cytotoxicity assay of CAR-NK cells prepared from BaEV-CD19 CAR-RVs and GALV-CD19 CAR-RVs.

Furthermore, the titer of BaEV-CD19 CAR-RV produced in large-scale industrial production was also tested. The results demonstrated that BaEV-CD19 CAR-RV is capable of maintaining high titers at 50 mL, 500 mL, and 1 L production volumes (**Figure 7A**, **Supplementary Table S4**). These findings indicate that the system can facilitate large-volume industrial production of BaEV-enveloped retroviral vectors. The results of the cytotoxicity assay demonstrated that CAR-NK cells prepared from BaEV-CD19 CAR-RVs exhibited greater cytotoxicity than did CAR-NK cells prepared from GALV-CD19 CAR-RVs (**Figure 7B**).

## 4 Discussion

Owing to their considerable potential for gene delivery to primary immune cells, BaEV-enveloped viral vectors have garnered increasing attention in cell and gene therapy. However, in the majority of current studies on BaEV envelopes, lentiviral vectors produced by transient transfection are used. This approach involves introducing all of the necessary genetic elements for virus production into cells through plasmid transfection, followed by the collection of virus-containing supernatant from the cell culture within a short period following transfection. The elevated cost of this process undoubtedly contributes to the overall expense of preparing viral vectors. In the clinic, the financial burden of these costs ultimately falls on the patient, which has resulted in high prices for cell and gene therapy products such as CAR-T and CAR-NK cells. In addition to the high cost, the transfection process inevitably generates residual plasmid DNA, which presents a challenge to the subsequent purification process and product quality control. In contrast, retroviral vectors can be produced by virus-producing cell lines. This method allows for the continuous harvesting of viral vectors in cell culture by simply transferring the target gene into a stable packaging cell line, which represents a significant advantage in large-scale industrial production. However, the cytotoxicity of BaEV envelope proteins represents a significant obstacle to the advancement of this system. In this study, we sought to produce BaEV envelope retroviral vectors by employing a stable packaging cell line-based approach. First, the impact of BaEV envelope proteins on cellular activity was investigated by knocking out the ASCT-1/2 receptor in packaging cell lines prior to the transfer of the BaEV envelope. This finding provides a potential strategy for the construction of stably packaged cell lines. On this basis, we further developed BaEV envelope-based retroviral vector-stably packed cell lines. The results demonstrate that a stable retrovirus-producing cell line can be established from this cell line and that the production of a series of BaEV-enveloped retroviral vectors, including BaEV-copGFP and BaEV-CD19 CAR, can be achieved. Notably, BaEV-CD19 CAR-RV can maintain a high production titer up to a culture volume of 1 L, indicating that the system can sustain a robust production capacity at the industrial scale. This approach has the potential to significantly reduce the cost of viral vector production, and even the costs of cell and gene therapy in practical applications.

Furthermore, the functionality of the retroviral vectors produced by this system was validated. The results of the cell transduction experiments revealed that the retroviral vectors produced by this cell line could significantly increase the gene transfer efficiency to T cells and NK cells. In particular, the BaEV-enveloped viral vectors generated from this cell line demonstrated a transduction positivity rate of up to 90% for NK cells, which was maintained even after fourfold dilution. In comparison, the transduction positivity rate of GALV-enriched viral vectors was approximately 20% at titers higher than the former. The cytotoxicity assay of CAR-NK cells provided further support for this result, and the transduced NK cells demonstrated sustained proliferative capacity until the 22nd day after transduction. This observation indicates that the retroviral vectors generated by this system do not impair the normal physiological functions of cells while introducing exogenous genes, thereby further demonstrating the great potential of this system for cell and gene therapy.

In conclusion, our research presents a novel production system for BaEV-enveloped retroviral vectors. This system addresses the challenges posed by BaEV in viral synthesis and is more suitable for industrial production. Compared with existing methodologies, the viral vectors produced by this system markedly increase the capacity for gene transfer in primary immune cells, particularly NK cells. On this basis, our system can facilitate the large-scale industrial production of BaEV-enveloped retroviral vectors through a comparable two-step methodology to that employed in the manufacture of GALV viral vectors based on the PG13 cell line. This will markedly increase the efficacy of NK cell gene transfer and the therapeutic efficacy of related therapies while greatly reducing the cost of immunotherapy, thereby facilitating the advancement of research on the biology of T cells, NK cells, or other cell types and the translation of various immune cell-based immunotherapies into clinical applications.

## Data Availability

The original contributions presented in the study are included in the article/**Supplementary Material**. Further inquiries can be directed to the corresponding authors.
