# Supplementary material for: Construction of stable packaging cell lines for large-scale industrial BaEV-enveloped retroviral vector production
Source: Front Immunol. 2025 May 26;16:1578660. doi: 10.3389/fimmu.2025.1578660 (PMC12146362; doi:10.3389/fimmu.2025.1578660)
Supplement: Supplementary file 1 [file DataSheet1.docx]

Supplementary Material

**Supplementary Table 1.** Viral titer produced by each HEK293T-gag-pol monoclonal cell line

|  | Positive Cells | Titer (TU/mL) |
| --- | --- | --- |
| Clone 1 | 62.8% | 1.54E+06 |
| Clone 2 | 8.38% | 2.05E+05 |
| Clone 10 | 9.69% | 2.37E+05 |
| Clone 14 | 6.71% | 1.64E+05 |
| Clone 19 | 15.8% | 3.87E+05 |
| Clone 28 | 44.8% | 1.10E+06 |

**Supplementary Table 2.** Viral titer produced by BaEV-Rless-57-copGFP and BaEV-WT-64-copGFP stably transduced cells

|  | Positive Cells | Titer (TU/mL) |
| --- | --- | --- |
| BaEV-Rless-57-copGFP | 2.42% | 9.68 E+05 |
| BaEV-WT-64-copGFP | 17.7% | 1.4 E+06 |

**Supplementary Table 3.** ASCT-1&2 gRNA sequences

| gRNA name | Sequence |
| --- | --- |
| ASCT-1-gRNA | AACAACGTAGAGCTCAACGC |
| ASCT-2-gRNA-1 | GAGGAATATCACCGGAACCA |
| ASCT-2-gRNA-2 | GGTTTACTCTTTGCCCGCCT |

**Supplementary Table 4.** Viral titer and NK cell transduction positivity of different types of BaEV-enveloped viral vectors produced on the basis of BaEV-PackRV

| CAR Target | Production volumes | Titer (TU/mL) | NK cell transduction efficiency |
| --- | --- | --- | --- |
| GD2 | 1L | 9.44E+05 | 85.93% |
| EGFR | 1L | 2.30E+06 | 84.32% |
| GPC3 | 1L | 1.62E+06 | 89.50% |


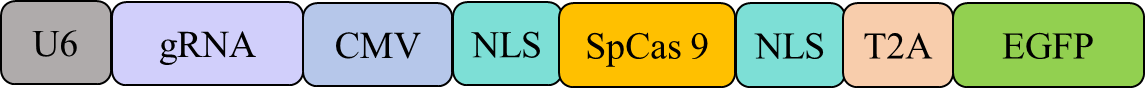


**Supplementary Figure 1.** The plasmid backbone structure for the PX458-ASCT-1/2-gRNA vectors


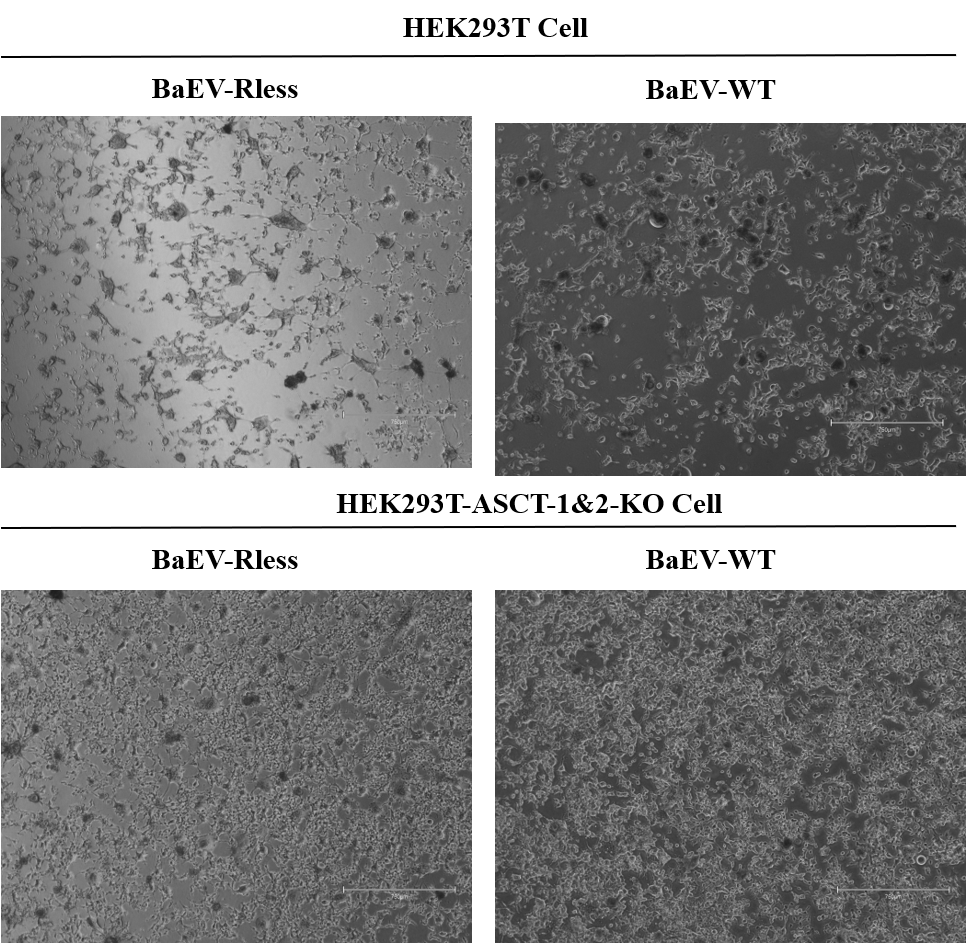


**Supplementary Figure 2.** Photographic documentation of HEK293T-gag-pol-ASCT-1&2-KO and HEK293T cell growth by microscopy after transfection with the BaEV-WT or BaEV-Rless plasmid.
